# Renal Arteriography and C-arm CT-Guided Ablation (RenACAGA) for Thermal Ablation of Challenging Renal Tumors

**DOI:** 10.1007/s00270-025-04039-1

**Published:** 2025-04-28

**Authors:** Maarten L. J. Smits, Niek Wijnen, Rutger C. G. Bruijnen, Willem M. Brinkman, Peter-Paul M. Willemse, Khalil Ramdhani, Maurits M. Barendrecht, Richard Meijer, Evert-Jan P. A. Vonken

**Affiliations:** 1 Department of Radiology, University Medical Center Utrecht, Utrecht, The Netherlands; 2 Department of Oncological Urology, University Medical Center Utrecht, Utrecht, The Netherlands

**Keywords:** C-arm CT, Local tumor recurrence, Microwave ablation, Renal arteriography, Renal cell carcinoma, Technical success

## Abstract

**Purpose:**

We present a technique that combines Renal arteriography with C-arm CT-Guided Ablation (RenACAGA) to improve tumor visualization, navigation and margin confirmation for percutaneous ablation of renal tumors.

**Materials and Methods:**

The RenACAGA technique was used for thermal ablation of challenging renal tumors (intraparenchymal or US-occult lesions). All patients treated with RenACAGA between January 1, 2022, and July 1, 2024, were retrospectively evaluated. Procedures were performed in the angiography suite, with catheterization of the renal artery for selective contrast infusion. C-arm CT and guidance software were used for tumor visualization and ablation needle placement. Pre- and post-ablation C-arm CTs were fused to assess ablation margins. Technical success and local tumor recurrence (LTR) rate were evaluated. Complications were graded according to the Common terminology criteria for adverse events (CTCAE) version 5.0.

**Results:**

Seven patients with 10 tumors were treated using the RenACAGA technique. All tumors were successfully identified, punctured and ablated (technical success 100%). During a median follow-up period of 8 months (range 7–25 months), no signs of tumor recurrence at the ablation site were observed (LTR rate 0%). One CTCAE grade 3 periprocedural complication was observed (urinary leakage through the needle tract), along with two CTCAE grade 1 complications (genitofemoral neuralgia (*n* = 1), and asymptomatic partial splenic infarction (*n* = 1)).

**Conclusion:**

The RenACAGA technique was successfully used for renal tumor ablation. Further studies are warranted to establish the potential benefits of this technique in terms of superior tumor visualization, targeting, ablation margin assessment, and combination with embolization.

**Graphical Abstract:**

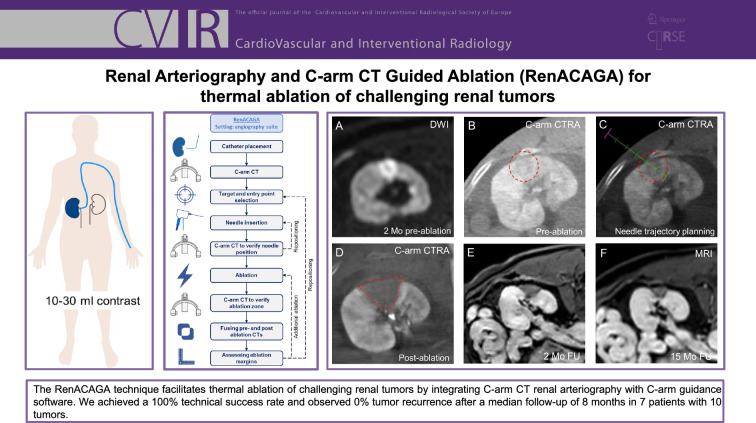

## Introduction

Percutaneous thermal ablation is a well-established minimally invasive alternative to kidney-sparing surgery for T1a renal cell carcinoma (RCC) [1, 2]. It can be performed using various techniques, including radiofrequency ablation (RFA), cryoablation, or microwave ablation (MWA) [3].

Ultrasound (US) and CT are commonly used for guiding renal ablation, as many renal tumors are easily detectable due to their exophytic location or distinctive echogenic or density differences. However, intraparenchymal tumors remain occult on both US and non-contrast CT, presenting a significant challenge in accurate localization and ablation [4, 5].

Using 3-phase intravenous contrast-enhanced CT-guidance requires high-volume contrast injections (80–110 ml each) for planning, needle position verification, and post-ablation assessment [6]. Needle repositioning or treating multiple tumors further increases contrast use, elevating the risk of contrast-induced acute kidney injury [7].

For liver tumors, selective intra-arterial contrast injection combined with CT imaging—known as CT hepatic arteriography—has emerged as a promising approach for thermal ablation [8–10]. CT hepatic arteriography significantly improves tumor visualization and ablation precision compared to traditional US-/CT-guided methods with intravenous contrast injection [8–12]. In this paper, we introduce the renal arteriography and C-arm CT-Guided Ablation (RenACAGA) technique for intraparenchymal or US-occult lesions. This method combines selective contrast injection via renal arteriography with C-arm CT guidance to enhance tumor visibility and improve the precision of thermal ablation.

## Materials and Methods

### Ethical Approval

The data used in this study were extracted from the ‘Minimally Invasive Thermal Ablation (MISTRAL) study’ database, a registry of all thermal ablation procedures performed at the University Medical Center Utrecht (Utrecht, The Netherlands). Permission was granted by the local institutional review board (No. 21/709), and the requirement for informed consent was waived.

### Patients

All consecutive patients with intraparenchymal or US-occult renal tumors scheduled for ablation using the RenACAGA technique between January 1, 2022, and July 1, 2024, were included in this retrospective analysis. Any histological subtype was included. All patients were referred for thermal ablation through a multidisciplinary tumor board.

### Treatment

**Fig. 1:**
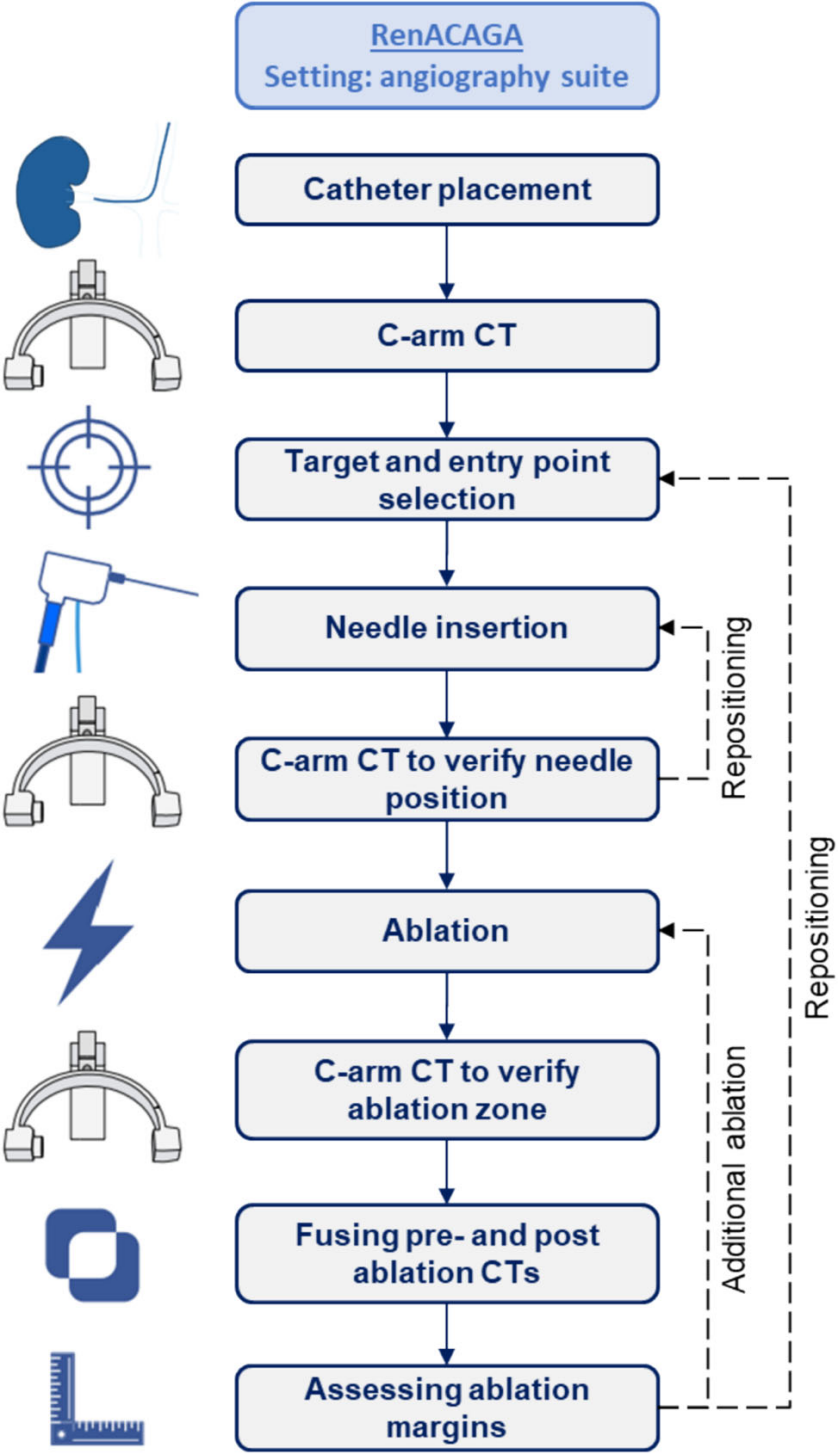
A flowchart of procedural steps involved in the RenACAGA technique. First, the femoral or radial artery was punctured, and a catheter was advanced to the renal artery. A C-arm CT with intra-arterial contrast was then performed to identify the target lesion and plan the needle trajectory by selecting the skin entry point and target. Next, the needle was advanced along the planned trajectory, followed by a C-arm CT scan to confirm accurate positioning. Once confirmed, ablation was initiated. Immediately after ablation, a C-arm CT scan was performed and fused with the pre-ablation scan to assess ablation margins. If the margins were inadequate, additional ablation was performed. Intra-arterial contrast was administered during all C-arm CT scans

A flowchart outlining the procedural steps involved in the RenACAGA technique is presented in Fig. 1.

#### Positioning

Treatment was performed in an angiography suite under general anesthesia to enable apnea during C-arm CT and needle placement. C-arm CT was performed using a monoplane C-arm system (Allura FD20 Xper, Philips, Best, the Netherlands) in propeller position with an ‘open trajectory rotation’ (240° rotation, 308 projections) and 10.4 s rotation time (XperCT HD fast setting). Positioning (prone or supine) depended on tumor location.

**Fig. 2:**
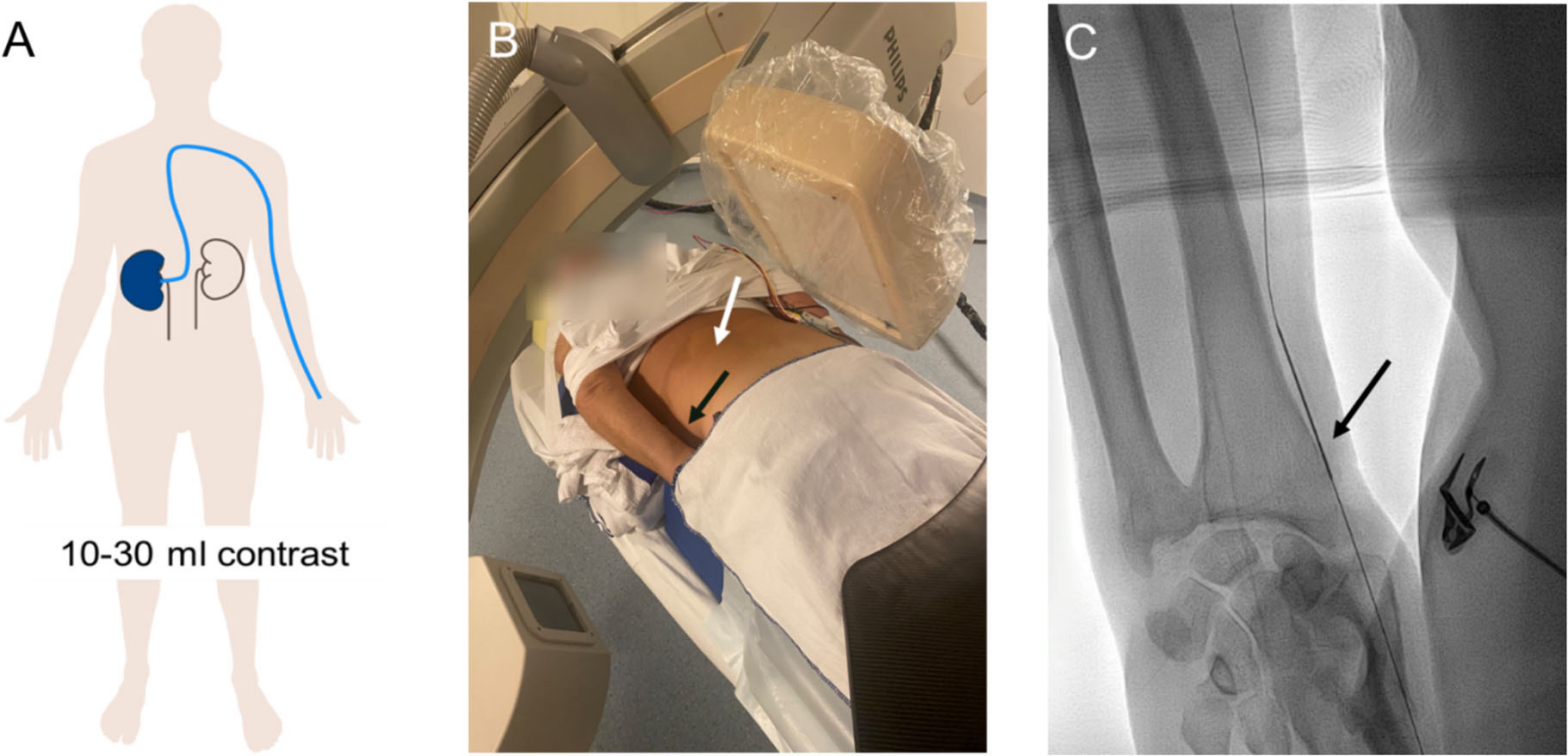
**A** Schematic representation of the catheterization of the right renal artery for intra-arterial contrast injection (10–30 ml) via radial access; **B** Patient positioning—The patient is in prone position with arms next to the body. Radial artery access site (black arrow), exposed flank to puncture the left kidney (white arrow); **C** Radial artery puncture site (black arrow) with a guidewire in position

*Prone position*: Arms placed alongside the body, palms up, allowing simultaneous radial artery and flank access for needle placement (Fig. 2).

*Supine Position*: The femoral artery was used for access, with one arm raised above the head for flank access.

#### Catheterization

Radial or femoral arterial access was achieved under ultrasound guidance. For radial access, a 5F sheath (Glidesheath™, Terumo) was inserted, followed by a 5F catheter (Performa, Merit) into the appropriate renal artery. For femoral access, a 6F sheath (Radifocus™, Terumo) was placed, followed by a 5F cobra catheter (Glidecath™, Terumo). Selective microcatheters (Progreat™, Terumo) were used in certain cases to access more specific branches of the renal artery.

#### C-arm CT with Renal Arteriography

**Fig. 3:**
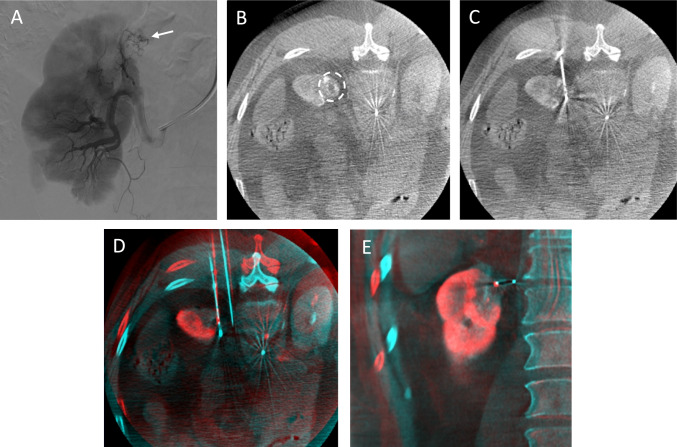
Example of RenACAGA ablation of a tumor in the upper pole of the left kidney. **A** Digital subtraction angiography of the left renal artery shows the target lesion (white arrow); **B** The target lesion (white circle) visualized with C-arm CT renal arteriography (C-arm CTRA); **C** Ablation needle in position; **D** and **E** Pre- and post-ablation C-arm CTRAs are fused with XperGuide software (Philips) in axial and coronal planes, showing adequate ablation margins

C-arm CT was performed during apnea and synchronized with intra-arterial contrast injection (renal arteriography) (Fig. 3). A 2:1 mixture of contrast agent (Visipaque 320 mg/ml) and saline was injected at a rate of 1.0–1.5 ml/s, with a 10-s scan delay. 10–30 ml of diluted contrast agent was used per C-arm CT.

#### Planning and Navigation

**Fig. 4:**
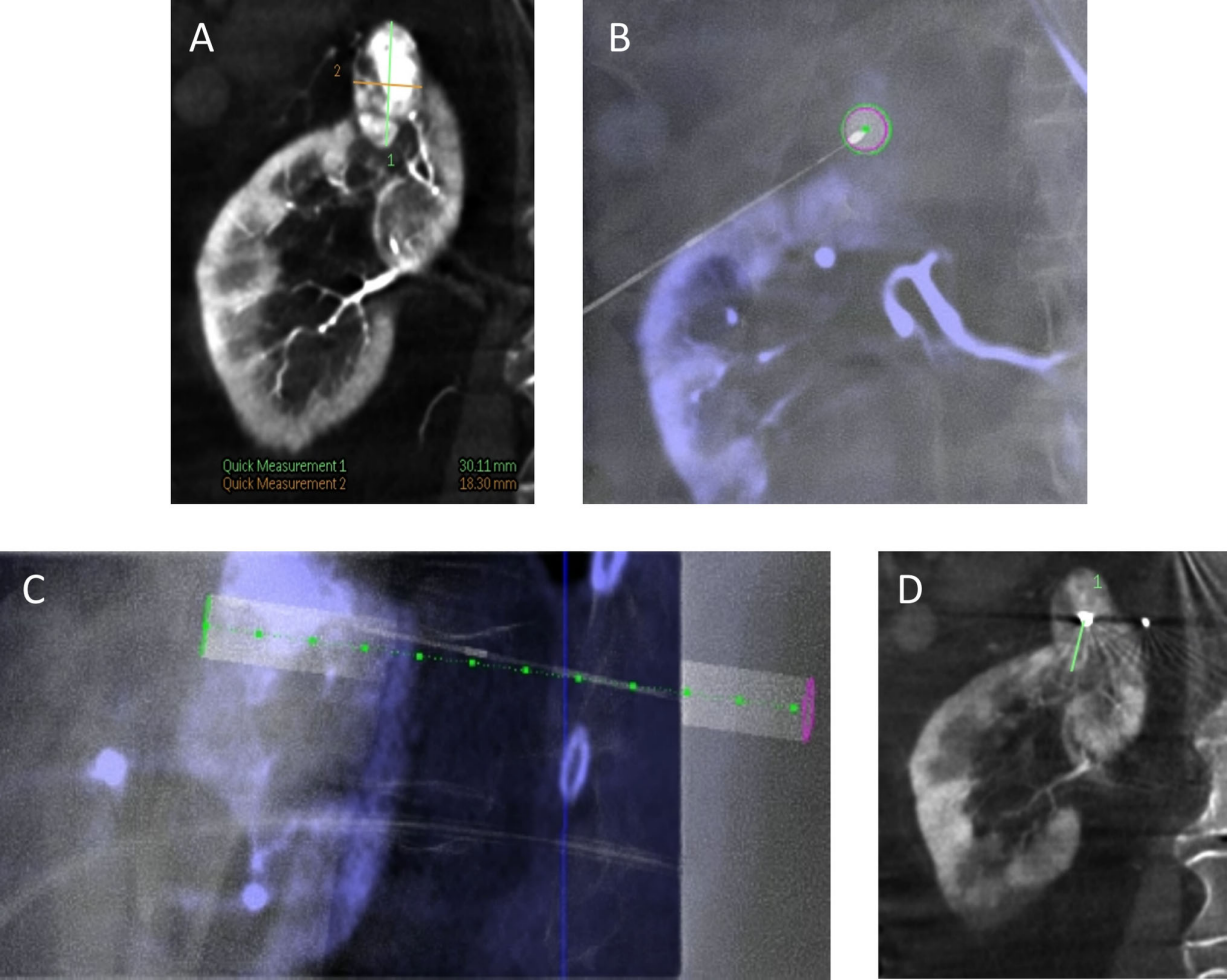
Example of navigation with XperGuide software (Philips). **A** Coronal view of the target lesion (largest diameter: 30 mm); **B** Entry point view, showing the entry point (pink circle) and target (green circle) projected on top of each other. The needle tip is aimed perpendicularly at the center of the planned needle trajectory. Next, the ablation needle is rotated 90 degrees to align directly with the projected needle trajectory (needle tip is kept at same position) to advance the needle in a straight line from entry point to target; **C** Perpendicular view to assess needle depth; **D** Needle is placed in center of target lesion

C-arm CT images were utilized to identify the target lesion and plan the trajectory for the microwave antenna (Emprint, Medtronic) or RFA applicator (Cooltip, Medtronic). Needle trajectory was planned using C-arm navigation software (XperGuide, Philips), which facilitated selection of the skin entry point and target lesion (Fig. 4). Using fluoroscopy, the needle was inserted along the projected path, and a confirmatory C-arm CT was obtained. If the needle position was incorrect, adjustments were made.

#### Thermal Ablation

Once the needle was connected to the generator (Emprint HP or Cooltip, Medtronic), ablation was initiated. Post-ablation C-arm CT with renal arteriography was performed to assess the ablation zone and identify potential complications. Pre- and post-ablation C-arm CTs were fused (XperGuide, Philips) to assess ablation margins (Fig. 3). In case of a minimum ablation margin < 5 mm, additional ablation was performed with needle repositioning if necessary. After ablation, the needle was withdrawn under tract ablation, and the catheter and sheath were removed. Hemostasis was achieved by applying compression at the arterial access site using an inflatable wristband (Safeguard Radial™, MeritMedical) for 2 h or with a vascular closure device (Angio-Seal™, Terumo) for the groin.

### Outcome Assessment

#### Primary Outcome

*Technical success*: Technical success was defined as the successful completion of the ablation procedure according to the RenACAGA technique, without switching to alternative modalities like US or conventional CT.

#### Secondary Outcomes

*Complications*: Adverse events occurring during or within 30 days post-procedure were documented by reviewing patient records. Complications were classified according to the Common terminology criteria for adverse events (CTCAE) v5.0 [13].

*Procedure duration*: Data on procedure duration were obtained from anesthesiology reports. "In-room time" was the time between patient entry and exit from the angiography suite, while "procedure time" was the time from arterial puncture to access site compression.

*Radiation dose*: The radiation dose received by the patient was measured as total dose–area product (DAP, Gy·cm^2^). To determine the effective dose (mSv), the total DAP was multiplied by a system-specific conversion factor of 0.38 mSv/Gy·cm^2^ [14].

*Local Tumor Recurrence (LTR)*: MRI or CT performed at 1, 3, and 6 months, and every 3–6 months thereafter, were reviewed to assess LTR. Any possible or definitive residual or local recurrence of the tumor at the site of the ablation zone was considered as LTR.

## Results

### Patients

**Fig. 5:**
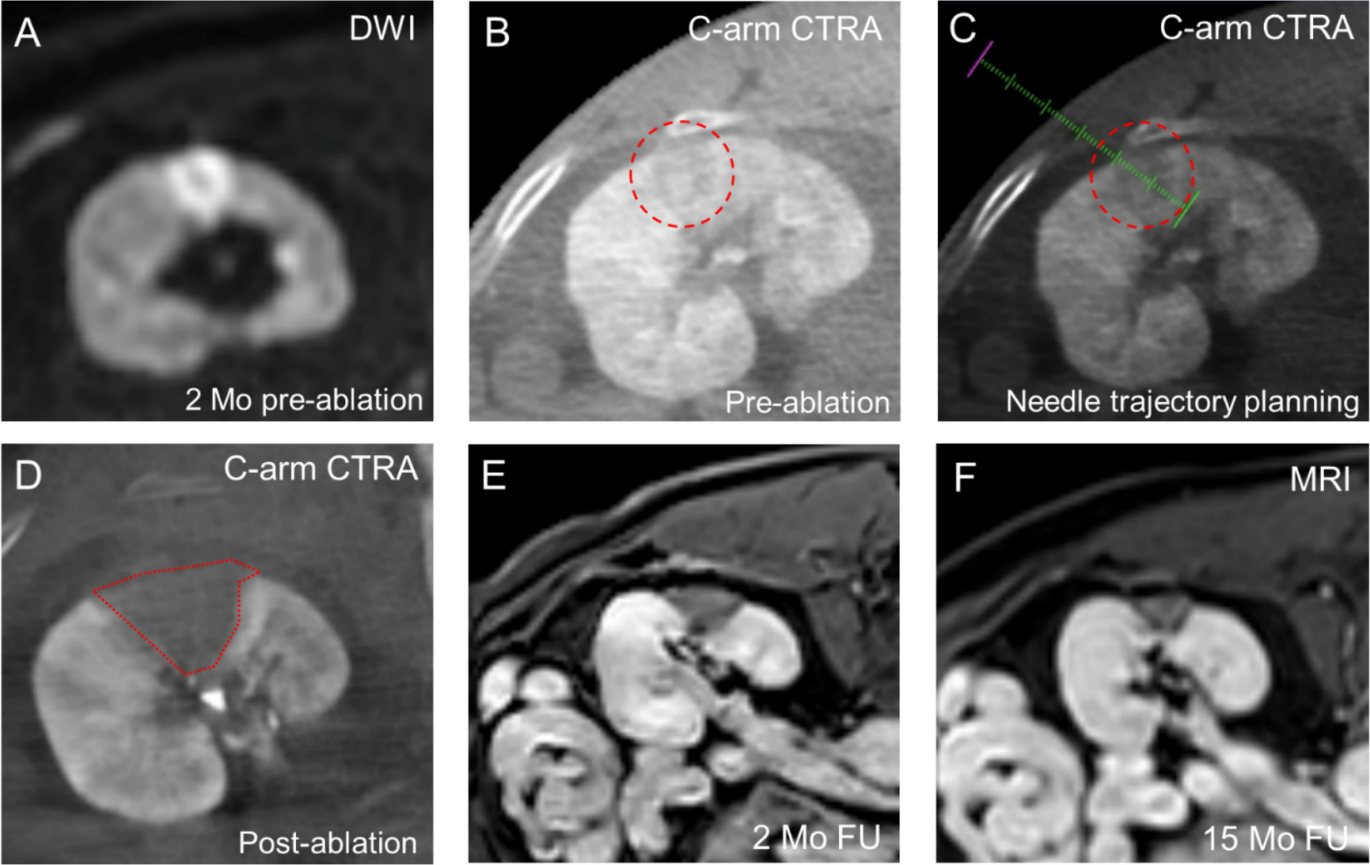
Example of a RenACAGA procedure of an intraparenchymal tumor (15 mm) in the left kidney. **A** The lesion is visualized on diffusion-weighted imaging (DWI) 2 months prior to the ablation procedure; **B** Intraprocedural C-arm CT renal arteriography (C-arm CTRA) depicts the target lesion (red circle); **C** Needle trajectory planning is performed using XperGuide software (Philips) to accurately target the tumor (red circle); **D** The ablation zone (outlined in red) after 2 min ablation at 100W is depicted with C-arm CTRA immediately after ablation; **E**, **F** Follow-up (FU) MRI (T1 with gadolinium) at 2 months and 15 months demonstrates no local tumor recurrence and a progressively shrinking ablation zone

During the inclusion period, 29 patients underwent renal tumor ablation, of which 7 patients (10 tumors) were treated with the RenACAGA technique and included for this analysis. The median tumor diameter was 20 mm (range 12–35 mm). 6/10 tumors were intraparenchymal and 4/10 tumors were occult on ultrasound. Figure 5 illustrates a RenACAGA procedure for an intraparenchymal lesion. Demographics are presented in Table 1.

**Table 1:** Demographics and tumor characteristics

Demographics		*n*	%/range
Patients		7	
Sex			
	Male	5	71%
	Female	2	29%
Age (median years, range)		64	28–81
BMI (median kg/m^2^, range)		28	22–39

Tumor characteristics		*n*	%
# of treated tumors		10	
Histological subtype			
	Papillary	6	60%
	Clear cell	1	10%
	Unclassified	3	30%
Tumor size (median diameter mm, range)		20	12–35
	< 20 mm	4	40%
	≥ 20 mm	6	60%

### Technical Success and Procedural Characteristics

All 10 tumors were successfully identified and ablated using the RenACAGA technique (technical success rate 100%) (Table 2). Six patients were treated in prone position for radial access and one patient in supine position for femoral access. MWA was conducted in 9/10 tumors at a median ablation power of 100W (range 75–150W) for a median ablation duration of 4.75 min (range 2.0–10.0 min). RFA was performed in 1/10 tumors at standard power settings for 6.0 min. Median effective dose was 46 mSv (range 20–104 mSv). In one patient, embolization with poly-vinyl alcohol particles (50-150 μm) (Contour™, Boston Scientific) was performed directly pre-ablation to reduce heat sink.

**Table 2:** Procedural characteristics

Procedural characteristics		*n*	%/range
Procedures		7	
Technically successful procedures		7	100%
Arterial access			
	Radial	6	86%
	Femoral	1	14%
Biopsy performed in the same procedure		5	71%
Ablation duration (median minutes, range)		4.75	2.0–10.0
In-room time (median minutes, range)		142	112–189
Procedure time (median minutes, range)		77	73–104
Effective dose (median mSv, range)		46	20–104
Ablation technology per treated tumor		**n**	**%**
Radiofrequency ablation		1/10	10%
Microwave ablation		9/10	90%
Median power (median Watt, range)		100	75–150

### Complications and Local Tumor Recurrence

One patient required temporary retrograde ureteric stenting for urinary leakage from an ablation tract and outflow obstruction caused by urethral stricture (CTCAE grade 3). The same patient developed genitofemoral neuralgia, despite hydrodissection between the kidney and psoas muscle, causing numbness in the groin and upper thigh without pain or muscular dysfunction (CTCAE grade 1). Additionally, an asymptomatic partial splenic infarction was observed as incidental finding on follow-up MRI (CTCAE grade 1), although its causal relationship with the ablation remains uncertain since neither the celiac trunk nor the splenic artery was catheterized (Table 3).

**Table 3:** Tumor recurrence and complications

Follow-up		*n*	%/range
Median imaging follow-up (months, range)		8	7–25
Follow-up modality			
MRI		4	57%
MRI + CT		3	43%
Patients with local tumor recurrence (concerning the ablated tumor)		0	0%

Complications		*n*	%
Uncomplicated procedures		6/7	86%
Complicated procedures		1/7	14%
CTCAE Grade 1			
	Genitofemoral neuralgia	1	14%
	Asymptomatic partial splenic infarction	1	14%
CTCAE Grade 3	Urinary leakage requiring temporary retrograde ureteric stenting	1	14%

During a median imaging follow-up of 8 months (range 7–25 months) no signs of local tumor recurrence were detected (LTR rate 0%).

## Discussion

In this case series, we present the RenACAGA technique for renal tumor ablation. This approach, which integrates renal artery catheterization with C-arm CT-guided tumor puncture, was successfully performed in all patients without major technique-related complications and no tumor recurrences during the follow-up period.

C-arm CT-guided ablation for renal tumors is well documented [15–18]. Ierardi et al. used image fusion between intraprocedural C-arm CT and preprocedural MRI/CT for renal tumor ablation [18]. While image fusion is technically feasible and overcomes limitations of C-arm CT guidance alone, inherent image distortion between pre- and intraprocedural imaging—due to differences in field of view, patient positioning, and resolution—limit fusion accuracy [19]. Since unenhanced C-arm CT poorly visualizes tumors and ablation zones, most studies used intravenous contrast. This often requires multiple high-volume contrast injections, increasing the risk of contrast-induced acute kidney injury [7].

Compared to intravenous contrast, renal arteriography offers superior differentiation between tumor and surrounding parenchyma, enhances ablation zone visualization, and requires significantly less contrast agent [9, 20]. Muglia et al. introduced CT renal arteriography (CTRA) for renal tumor ablation [20]. A drawback of CTRA is patient transfer between the angiography suite (catheterization) and CT room (ablation) in centers without a hybrid angiography-CT setup, increasing the risk of catheter dislodgment and contamination [8]. In contrast, C-arm CT guidance allows the entire procedure to be performed in the angiography suite, eliminating patient transfer, enabling immediate embolization in cases of post-ablation hemorrhage, and improving logistics [11].

The RenACAGA technique offers several other distinct advantages. Standard C-arm puncture software (e.g., XperGuide, Philips) facilitates accurate off-plane puncturing and improves ablation margin assessment through pre- and post-ablation C-arm CT fusion. Additionally, the method allows for enhanced tumor ablation by performing bland embolization using poly-vinyl alcohol particles immediately prior to ablation, reducing the heat sink effect [21–23]. This can be particularly beneficial for hypervascular renal tumors [24, 25].

The RenACAGA technique has some limitations. Arterial access carries risks such as bleeding, dissection, or thromboembolism [26]. Other limitations are the requirement for general anesthesia to induce apnea during C-arm CT and needle placement, increased radiation exposure from real-time fluoroscopy during needle placement and extended procedure time. One patient experienced perirenal urinary leakage after ablating three tumors in one session with high-power settings. This may have caused excessive heating of the urinary collecting system, leading to ureteral stricture and outflow obstruction. Caution is advised when performing MWA for multiple tumors without thermoprotective measures.

Limitations of this study include its retrospective design, small sample size, and short follow-up, which limits the broader applicability and generalizability of the results. Nevertheless, this study serves as a proof-of-concept, demonstrating the feasibility, safety, and effectiveness of the RenACAGA technique. Although RenACAGA was used for challenging renal tumors only (intraparenchymal or US-occult lesions), it is now increasingly used at our center for non-challenging RCC as well because of the aforementioned advantages.

In conclusion, we presented the renal arteriography and C-arm CT-Guided Ablation (RenACAGA) technique for treating renal tumors. It has demonstrated to be technically feasible, effective, and associated with a low complication rate. Further studies need to investigate if this technique improves tumor visualization, targeting accuracy, and ablation margin assessment.
